# Supplementary figures and images for: Beyond the Hypercube: Evolutionary Accessibility of Fitness Landscapes with Realistic Mutational Networks
Source: PLoS Comput Biol. 2016 Dec 9;12(12):e1005218. doi: 10.1371/journal.pcbi.1005218 (PMC5147777; doi:10.1371/journal.pcbi.1005218)

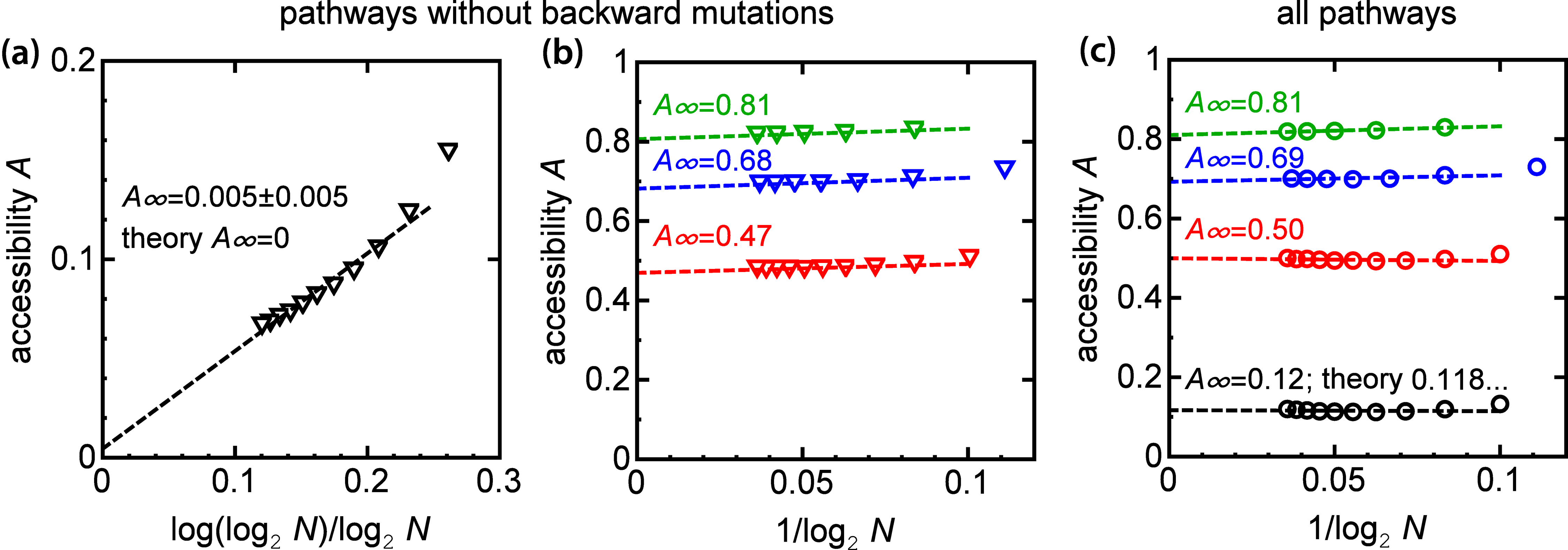

Supplement: S1 Fig — (a) Asymptotic estimate of accessibility A versus the log(log2 N)/log2 N for K = 2 when only shortest pathways are considered. The logarithmic correction to the inverse of log2 N comes from mathematical arguments [31]. (b,c) Plots of accessibility A versus the inverse of log2 N for different K (2 = black, 4 = red, 8 = blue, and 16 = green) and for pathways without backward mutations (panel b) and for all pathways (panel c). The dashed lines are linear fits to the data subset. Only landscapes with N > 210 have been used to estimate A∞. Statistical errors (s.e.m.) are below 0.01 for all A∞. (TIF) [file pcbi.1005218.s001.tif]

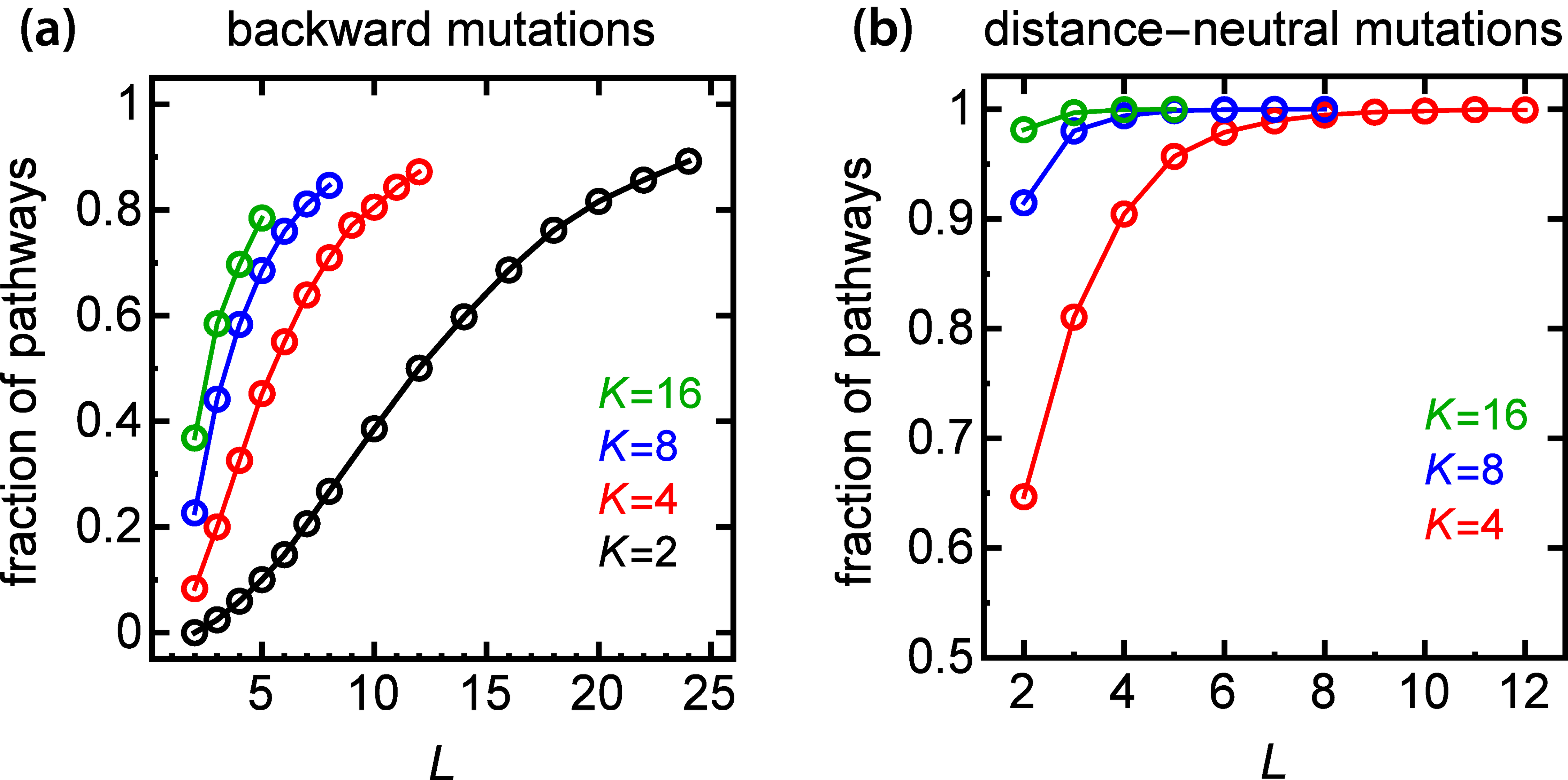

Supplement: S2 Fig — (a) Average fraction of pathways that contain at least one backward mutation as a function of genotype length L, and for different K. (b) Analogous to panel a, but for distance-neutral mutations. By definition, distance-neutral mutations do not exist for K = 2 and hence there is no corresponding line in the plot. (TIF) [file pcbi.1005218.s002.tif]

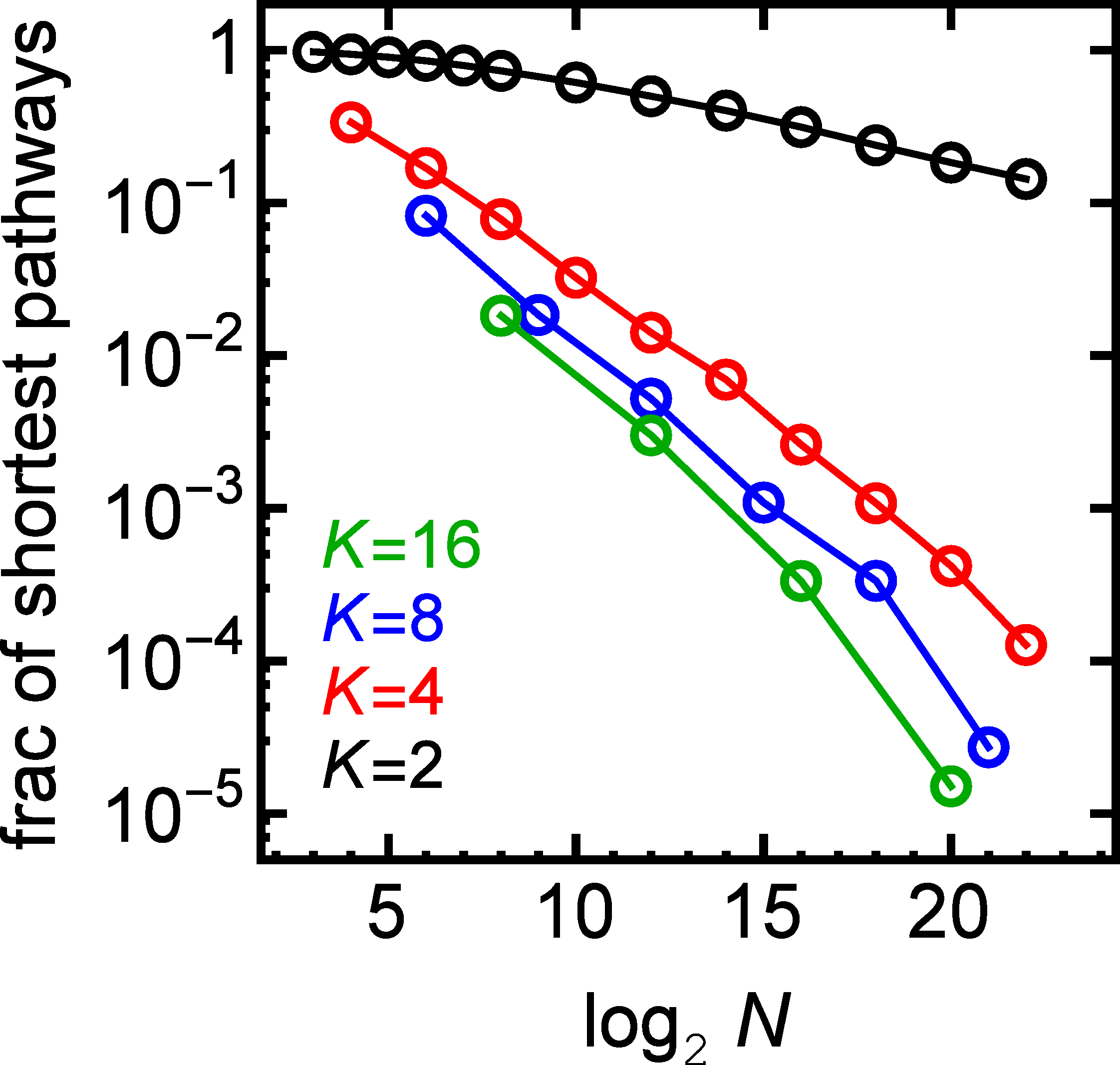

Supplement: S3 Fig — The average fraction of shortest pathways as a function of number of genotypes N, and for different K. The median of the fraction of shortest pathways appears to tend to zero for K > 2 in the large-N limit. (TIF) [file pcbi.1005218.s003.tif]

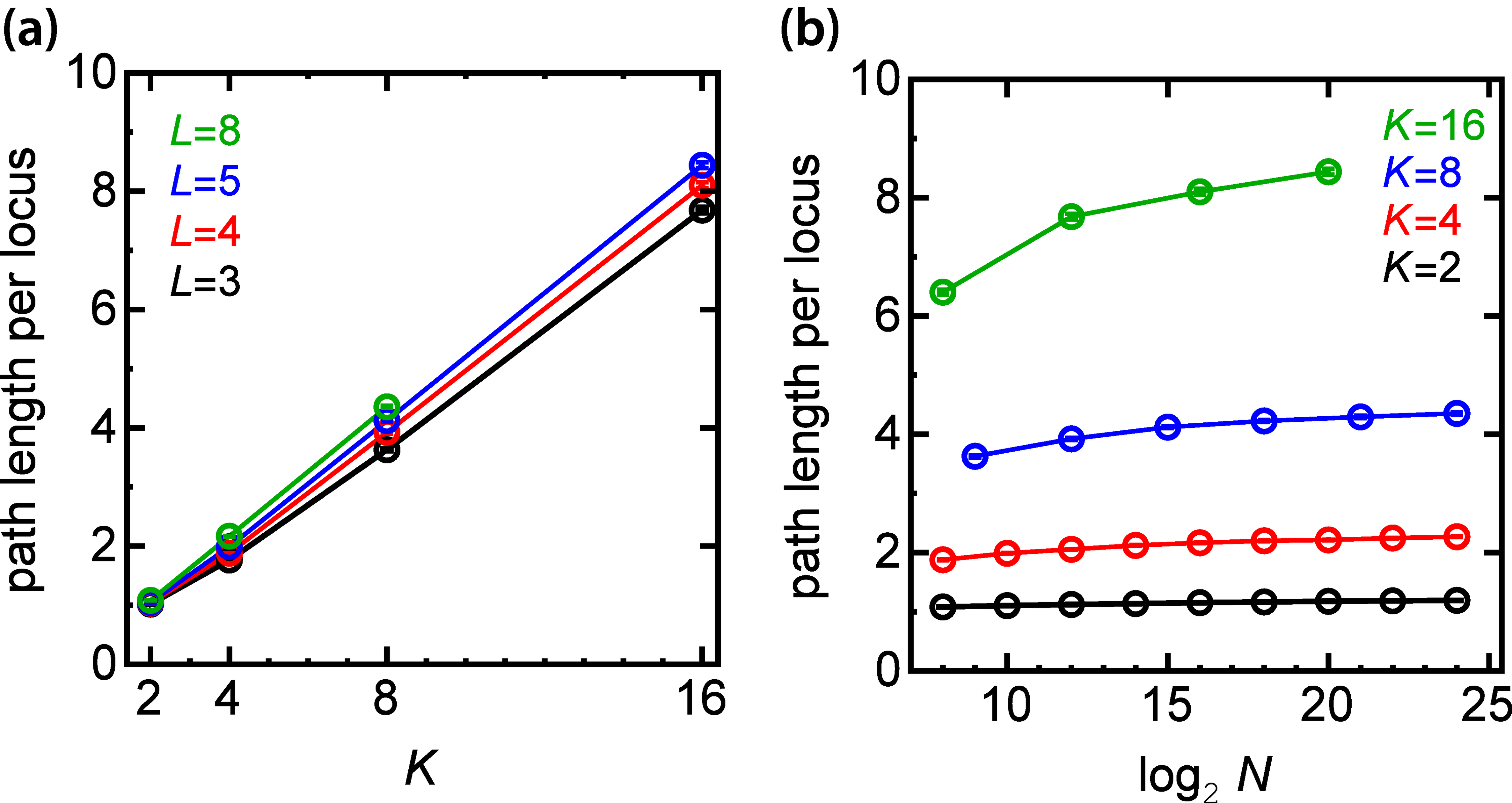

Supplement: S4 Fig — (a) The normalized pathway length (number of mutational steps divided by L) as a function of K, for different values of L. (b) The normalized length as a function of log2 N for different values of K. Except from K = 16 the normalized length reaches a plateau within the investigated range of N. (TIF) [file pcbi.1005218.s004.tif]

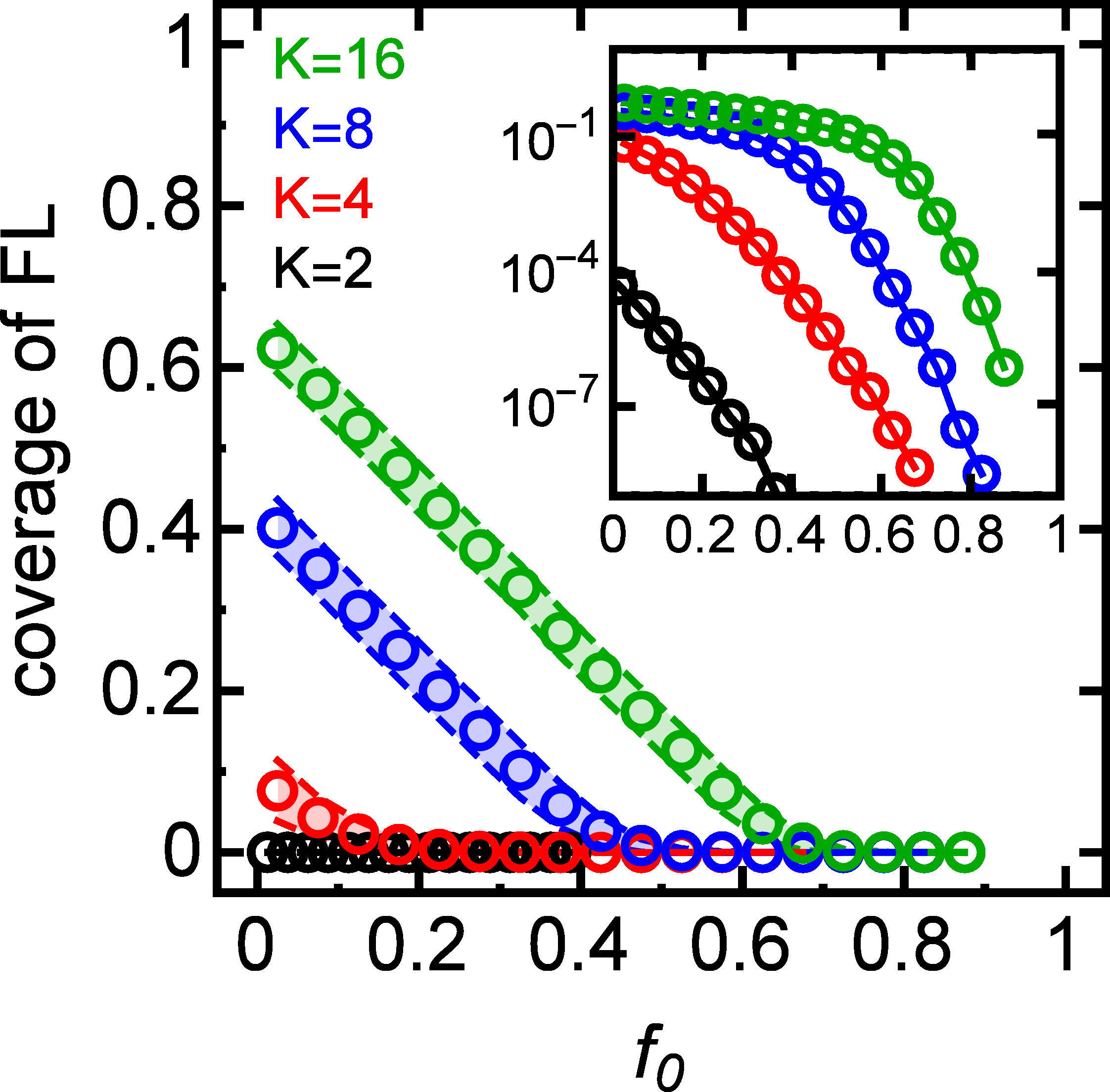

Supplement: S5 Fig — Average coverage of fitness landscapes with different number of coding units K (black, red, blue, green) and a fixed number of genotypes (N = 224 for K = 2, 4, 8 and N = 220 for K = 16) as a function of initial fitness f0. Inset: the same data in the log-linear scale. Shaded area corresponds to one standard deviation (accessible FLs only). (TIF) [file pcbi.1005218.s005.tif]

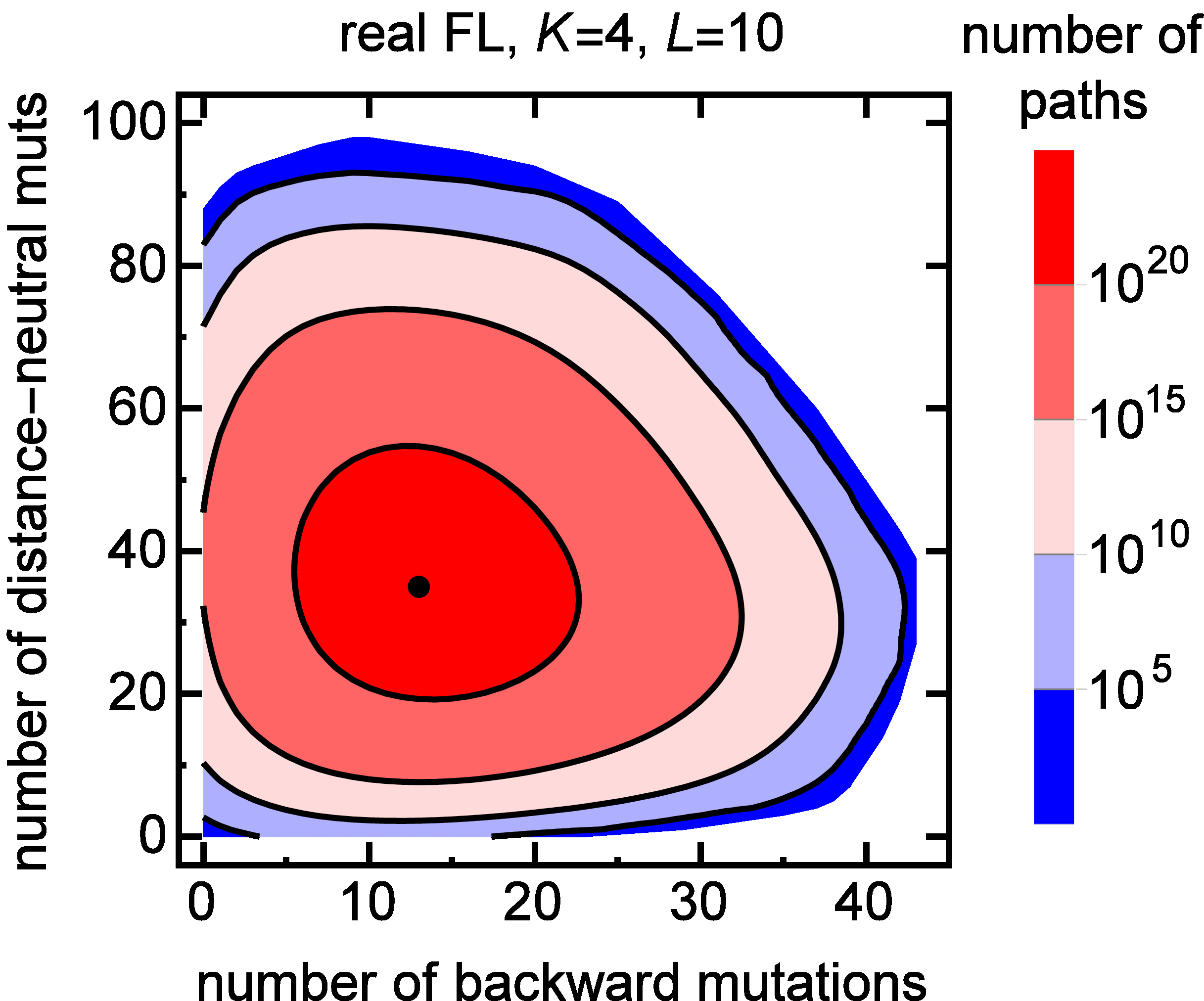

Supplement: S6 Fig — Number of accessible pathways from the antipodal to the best-fit genotype for the full experimental FL as a function of the number of backward and distance-neutral mutations. The maximal number of pathways (black dot) is approximately 6.45 × 1021. The histogram was obtained by exhaustive enumeration of all pathways using the algorithm described in Methods. (TIF) [file pcbi.1005218.s006.tif]

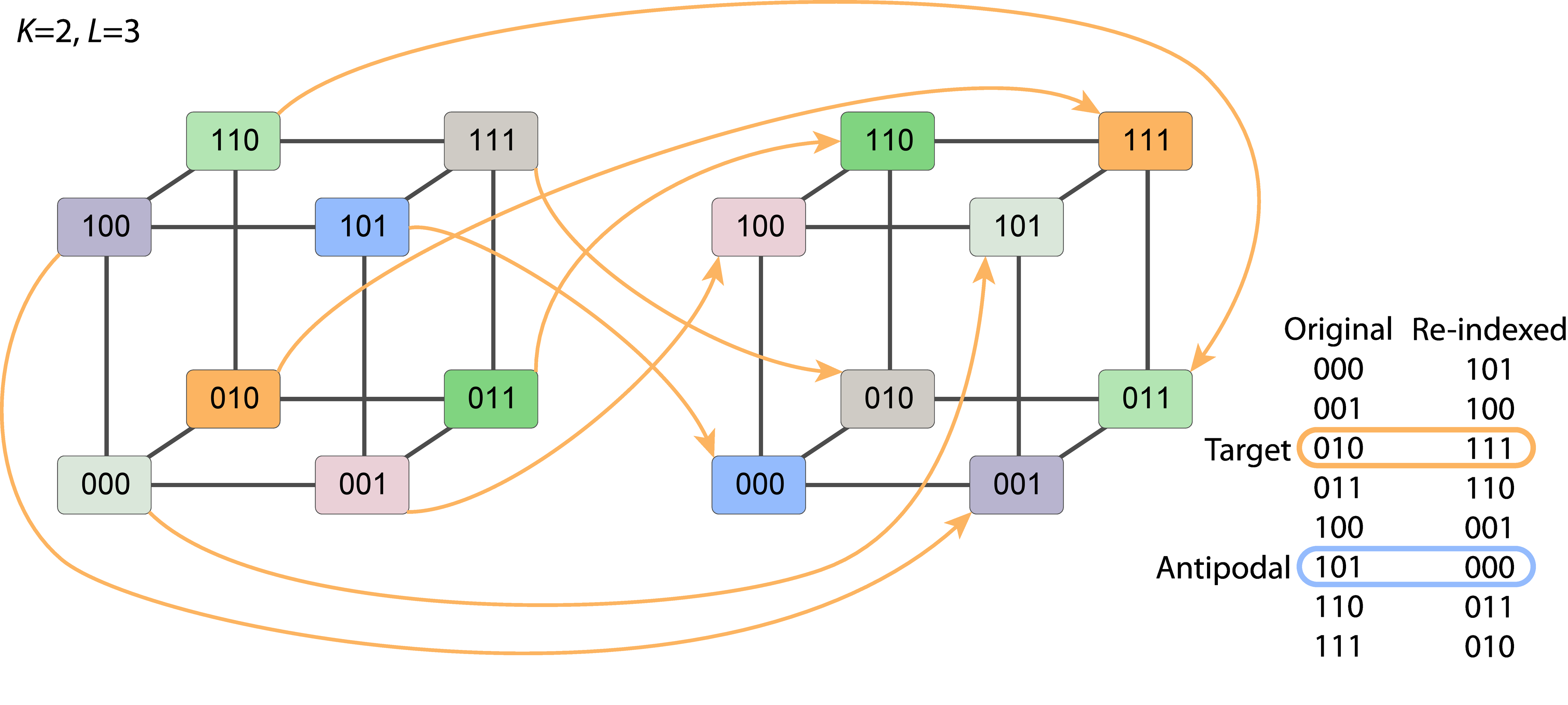

Supplement: S7 Fig — Example of original (left) and re-indexed (right) fitness landscapes with target (best-fit) and antipodal genotypes highlighted. The aim of re-indexing is to reassign genotypes in isomorphic manner (the structure of the fitness landscape is unchanged), such that the target genotype is assigned sequence 11…1 and the antipodal genotype 00…0. For each genotype the original sequence and the re-indexed sequence are highlighted with the same colour and linked with an arrow. (TIF) [file pcbi.1005218.s007.tif]
