# Supplementary material for: Beyond the Hypercube: Evolutionary Accessibility of Fitness Landscapes with Realistic Mutational Networks
Source: PLoS Comput Biol. 2016 Dec 9;12(12):e1005218. doi: 10.1371/journal.pcbi.1005218 (PMC5147777; doi:10.1371/journal.pcbi.1005218)
Supplement: S1 Table — Properties of the two fitness landscapes generated from the experimental data sets discussed in the main text [39, 42]. Randomized ensembles of FLs preserve fitness values, but not fitness-to-distance correlations. (PDF) [file pcbi.1005218.s008.pdf]

10k sub-FLs generated from DNA-protein binding affinities (Rowe et al.)

	K=4, L=5		K=2, L=10	
	Experimental	Randomized	Experimental	Randomized
accessibility A	0.86	0.51	0.67	0.13
⟨coverage of FL⟩*	28%	8%	26%	3%
⟨pathway length⟩	16.8	10.0	15.4	11.1
⟨forward mutations⟩	43%	57%	82%	95%
⟨distance − neutral mutations⟩	44%	37%	0%	0%
⟨backward mutations⟩	13%	7%	18%	5%
⟨pathway number⟩	1.2×10^{10}	1.9×10^{3}	2.3×10^{6}	19
⟨log10(pathway number)⟩	5.4	2.1	3.9	0.8

1k sub-FLs generated from RNA-protein binding affinities (Guenther et al.)

	K=4, L=3		K=2, L=6	
	Experimental	Randomized	Experimental	Randomized
accessibility A	0.95	0.59	0.78	0.20
⟨coverage of FL⟩	42%	24%	44%	19%
⟨pathway length⟩	6.7	5.3	7.1	6.4
⟨forward mutations⟩	52%	61%	92%	97%
⟨distance − neutral mutations⟩	41%	35%	0%	0%
⟨backward mutations⟩	7%	4%	8%	3%
⟨pathway number⟩	1.8×10^{3}	58	131	6.1
⟨log10(pathway number)⟩	2.2	1.2	1.6	0.6

*) The average ⟨…⟩ was restricted to accessible FLs: for each such FL we first calculated the corresponding quantity, and then averaged it over all accessible FLs. Statistical errors (s.e.m.) are at most +/-1 of the last digit.
